# Poor Sleep Quality Worsens Static and Dynamic Balance Control in Individuals With Chronic Low Back Pain: A Cross-Sectional Study

**DOI:** 10.1155/prm/5224748

**Published:** 2025-02-24

**Authors:** Daniel K. Y. Zheng, Zhihan Sun, Jeremy R. Chang, Frank F. Huang, Yilin Liu, Siying Yu, Jinlong Wu, Zimeng Wang, Arnold Y. L. Wong, Xueqiang Wang

**Affiliations:** ^1^Department of Rehabilitation Sciences, The Hong Kong Polytechnic University, Hong Kong, China; ^2^Department of Sport Rehabilitation, Shanghai University of Sport, Shanghai, China; ^3^Department of Sport Medicine, Sichuan Province Orthopedic Hospital, Chengdu, China; ^4^College of Physical Education, Southwest University, Chongqing, China; ^5^Beijing Key Laboratory of Fundamental Research on Biomechanics in Clinical Application, School of Biomedical Engineering, Capital Medical University, Beijing, China; ^6^Research Institute for Smart Ageing, The Hong Kong Polytechnic University, Hong Kong, China; ^7^Rehabilitation Medicine Center, The Second Affiliated Hospital and Yuying Children's Hospital of Wenzhou Medical University, Wenzhou, China

**Keywords:** anxiety, disability, low back pain, postural balance, sleep disorder

## Abstract

**Objective:** To investigate the influence of sleep quality and associated factors on balance control in individuals with chronic low back pain (CLBP).

**Methods:** 85 participants (mean age 33.2 ± 12.5 years) with CLBP were recruited. Physical and emotional well-beings were evaluated using a battery of questionnaires. Sleep quality over the last month was assessed using the Pittsburgh Sleep Quality Index (PSQI). Participants were dichotomized into the good sleep quality (GSQ) and poor sleep quality (PSQ) groups if their PSQI scores were ≤ 5 and > 5, respectively. Balance control was measured using the one-leg stance with eyes closed and Y-balance test.

**Results:** The GSQ group included 37 participants, while the PSQ group comprised 48 participants. After controlling for confounds (including gender, age, disability, anxiety, depression, and fear avoidance beliefs), participants with PSQ displayed significantly poorer performance in the one-leg stance with eyes closed and lower normalized posteromedial, posterolateral, and composite scores of the Y-balance test compared with participants with GSQ. Additionally, sleep quality accounted for 16.9%–24.9% of the variance in balance control, while age explained an additional 5.2%–13.2% of the variance. Additionally, higher levels of physical disability and anxiety were associated with poorer balance control.

**Conclusions:** Individuals with concurrent CLBP and PSQ exhibit significantly worse balance control than those with CLBP alone. Future studies should investigate whether improving sleep quality, physical disability, and anxiety can enhance balance in individuals with CLBP.

## 1. Introduction

Low back pain (LBP) is a significant global public health issue, affecting over 80% of individuals at some point in their lives [1, 2]. Approximately 20.1% of LBP cases progress to chronic LBP (CLBP), characterized by pain lasting longer than 12 weeks [3, 4]. Moreover, sleep disturbances have been frequently observed in individuals with CLBP. Previous studies have reported that 50%–89% of individuals with CLBP experience some forms of sleep problems (e.g., difficulties in initiating or maintaining sleep), resulting in a diminished quality of life and substantial costs to society [5–8].

Previous studies have established a prospective and bidirectional association between poorer sleep quality and more severe CLBP [9, 10]. Specifically, individuals with poor sleep quality (PSQ) are more likely to develop CLBP compared to those with good sleep quality (GSQ) [11]. Oliveira et al. found that PSQ at baseline predicted higher LBP-related disability after 6 months in older adults with LBP [12]. Furthermore, compared to asymptomatic controls, individuals with CLBP reported significantly higher Pittsburgh Sleep Quality Index (PSQI) scores, indicating poorer sleep quality [13].

Balance control relies on an intricate integration of the central nervous system with visual, proprioceptive, and vestibular inputs [14, 15]. Previous research has indicated that sleep deprivation disrupted visual, proprioceptive, and vestibular functions in healthy individuals [16–21], which can have a detrimental impact on balance control [18, 22]. PSQ is known to be associated with impaired balance control in both healthy adults and individuals with fibromyalgia [23–25]. PSQ may also lead to certain psychological conditions (such as depression and anxiety) that are commonly experienced by individuals with CLBP [11, 26, 27]. Previous research revealed that anxiety, depression, and fear avoidance beliefs share common pathways that mediate balance control [28, 29]. As such, further investigation is necessary to uncover the impact of these concomitant factors on balance control in individuals with CLBP.

Given this background, the current study aimed to examine the relative influence of sleep quality and various physical and psychological factors on balance control in adults with CLBP. We hypothesized that (1) individuals with CLBP and concomitant PSQ would display inferior balance control compared to those with CLBP and GSQ and (2) other factors (such as depression and anxiety) would also have negative effects on balance control in adults with CLBP. The findings would contribute to a better understanding of the determinants of compromised balance control in individuals with CLBP and provide insights for the comprehensive management of CLBP cases.

## 2. Materials and Methods

### 2.1. Sample Size Calculation

Previous studies revealed that the effect sizes for detecting the deficits in static and dynamic balance in healthy adults with GSQ or PSQ ranged from 0.54 to 1.15 [24, 25]. Assuming that individuals with CLBP and GSQ or PSQ displayed a similar difference in balance control, 35 participants with GSQ or 35 participants with PSQ were needed with an effect size of 0.54, a statistical power of 0.95, and an alpha level of 0.05 as estimated by the G∗Power software (Version 3.1.7, Heinrich Heine University Dusseldorf, Germany).

### 2.2. Participants

The study methods and results were reported following the STrengthening the Reporting of OBservational studies in Epidemiology (STROBE) statement for cross-sectional studies [30]. This cross-sectional study was approved by the Ethics Committee of Shanghai University of Sport (approval number: 2018069). Participants were recruited from the campus, school hospital, and nearby communities through e-posters on a social mobile application, WeChat, using a convenience sampling approach. All participants provided written informed consent and underwent eligibility screening. The inclusion criteria were (1) adults aged between 18–65 years with LBP located between the 12^th^ rib and buttocks, with or without leg pain [31]; (2) a LBP duration for more than 12 weeks [32]; and (3) an average pain intensity of at least 2 points out of 10 on an 11-point Numerical Rating Scale (NRS) in the last seven days, where 0 means no pain and 10 means the worst imaginable pain [33, 34]. Exclusion criteria included (1) the inability to complete the assessments of this study; (2) current pain intensity exceeding eight points on the NRS [35]; (3) the presence of other musculoskeletal conditions (e.g., fractures, multiple joint pain, tumors, spinal stenosis, or arthritis in the lower limbs); (4) a history of spine or lower extremity surgery; (5) pregnancy; or (6) uncorrected visual or vestibular disorders.

### 2.3. Procedures

**Figure 1:**
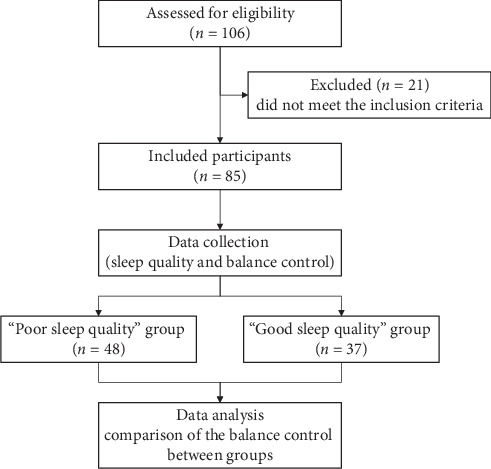
Study flowchart.

Participants were instructed to complete a set of questionnaires assessing their demographics, LBP intensity, LBP-related disability, and psychological well-being. This was followed by self-reported sleep quality assessments and balance tests (Figure 1).

#### 2.3.1. Demographic and Clinical Characteristics

Pain intensity, including current pain, average pain, and maximum pain intensity over the past week, was measured using the 11-point NRS [36]. LBP-related disability was assessed using the Chinese version of the Roland-Morris Disability Questionnaire (RMDQ), with higher scores indicating more disability [37]. Anxiety and depression levels over the last seven days were measured by the Chinese version of the 20-item Zung Self-Rating Anxiety Scale (SAS) and the 20-item Zung Self-Rating Depression Scale (SDS), with higher scores indicating more anxiety and depression, respectively [38]. Fear avoidance beliefs were assessed using Chinese version of the Fear-Avoidance Beliefs Questionnaire (FABQ), which includes subscales for physical activities (FABQ-P) and work activities (FABQ-W) [39]. Higher scores indicate more severe fear-avoidance beliefs toward physical or occupational activities. Additionally, each participant's leg length was determined by measuring the distance between the anterior superior iliac spine and the most prominent part of the ipsilateral medial malleolus in a supine position.

#### 2.3.2. Sleep Quality Assessment

The Chinese version of the PSQI was used to evaluate the participants' sleep quality. The PSQI is a reliable and valid measure of sleep quality in the last month [40, 41]. The total score ranges from 0 to 21, with lower scores indicating better sleep quality. A cutoff score of five was used to distinguish between individuals with GSQ and PSQ, with a sensitivity of 89.6% and a specificity of 86.5% [41]. As such, our participants were dichotomized into GSQ (PSQI scores ≤ 5) and PSQ (PSQI scores > 5) groups.

#### 2.3.3. One-Leg Stance With Eyes Closed (OLS-C)

The OLS-C was used to assess the static balance ability of the participants. Prior research has shown excellent inter-rater reliability of this test in assessing individuals with LBP (intraclass correlation coefficients (ICC) = 0.88–1.0) [35]. Furthermore, Sung and Leininger (2015) found that the OLS-C was more effective than the eyes-open condition in detecting balance deficits in individuals with LBP [42]. Participants were instructed to stand on a rigid platform with bare feet and their hands on their waists. Upon the “start” cue, the participant closed the eyes and raised one leg simultaneously. The participant was required to maintain balance for up to 120 s [43], while a timer recorded the duration. The timer stopped if the supporting foot moved, the free leg touched the ground or the supporting leg, or if either hand was taken off the waist [44]. Each leg performed three trials, with the order randomized by a coin toss. A 30 s break was given before switching to the other leg to prevent fatigue [45, 46]. Participants were allowed to practice twice on each leg before the actual data collection. The average duration (in seconds) from both legs was used for the analysis, with a longer duration indicating better static balance performance.

#### 2.3.4. Y-Balance Test (YBT)

The lower quarter YBT (FunctionalMovement.com, Danville, VA) was employed to assess the participants' dynamic balance ability. This test has demonstrated excellent inter-rater reliability (ICC = 0.99–1.0) in individuals with CLBP [34]. During this test, participants were instructed to stand on one leg on a fixed platform, place their hands on their waist, and maintain balance, while the foot of contralateral leg pushed an indicator as far as possible in three directions: anterior, posteromedial, and posterolateral. To ensure consistency and reproducibility, the test sequence followed a specific order, starting with the anterior direction and then moving to the posteromedial and posterolateral directions. The trial was considered invalid if the participant (1) failed to maintain balance on one leg or return the reaching foot to the starting position; (2) failed to keep their hands on their waist; (3) used the ground or the indicator for foot support; or (4) kicked the indicator [25, 47]. Three trials were conducted with a 10 s break between each trial, and the average distance was calculated for each direction. The order of legs was randomized using a coin toss, with a 30 s break before switching to another leg to minimize the risk of fatigue [47, 48]. Prior to the formal trials, participants were allowed to practice twice in each direction. The reaching distance in centimeter was measured and then normalized to the length of the reaching leg using the formula: (reaching distance/reaching leg length) × 100% [49]. A composite score was calculated by averaging the normalized scores in three directions (anterior, posteromedial, and posterolateral). The average normalized anterior, posteromedial, posterolateral, and composite scores from both legs were used for the analysis, with higher scores indicating better dynamic balance performance.

### 2.4. Statistical Analysis

For demographic and clinical characteristics, the chi-square tests were utilized to compare between-group differences in categorical variables. The normality of continuous variables was assessed by the Kolmogorov–Smirnov test. Independent *t*-tests were employed to compare differences between groups for parametric variables, while Mann–Whitney *U* tests were used for nonparametric variables. For balance performance, analysis of covariance (ANCOVA) was used to identify the difference between groups after controlling for various confounding demographic and clinical characteristics that showed between-group differences (with *p* < 0.1). Separate forward stepwise regression models were performed to identify factors that were independently related to OLS-C and normalized YBT scores. Variables entered were the abovementioned confounding demographic and clinical characteristics. These variables were entered into the model at a significance level of *p* ≤ 0.05, and they were removed from the model if their significance level reached *p* ≥ 0.1 [50]. Statistical analyses were performed using the SPSS 29.0 (IBM Corp, Armonk, NY, USA) with the significance level set at *p* < 0.05.

## 3. Results

### 3.1. Participants' Characteristics

A total of 85 participants with CLBP, including 37 males and 48 females (33.2 ± 12.5 years), were recruited (Table 1). Among them, 48 participants (56%) reported PSQ, while 37 participants (44%) reported GSQ. The mean PSQI score in the PSQ group (9.2 ± 3) was significantly higher than that in the GSQ group (3.7 ± 1.4) (*p* < 0.001). No significant differences were observed in various demographic variables between the two groups. However, compared to the GSQ group, the PSQ group exhibited higher scores on RMDQ (*p*=0.003), SAS (*p* < 0.001), SDS (*p* < 0.001), FABQ (*p* < 0.001), FABQ-P (*p*=0.014), and FABQ-W (*p*=0.002).

### 3.2. Static and Dynamic Balance

**Figure 2:**
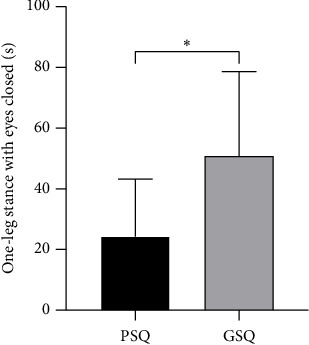
The results of one-leg stance test. *Note:* Data are expressed as the mean ± standard deviation; GSQ, good sleep quality; PSQ, poor sleep quality. ⁣^∗^*p* < 0.05.

**Figure 3:**
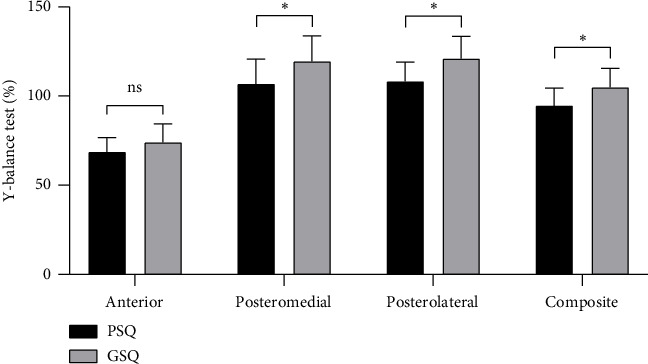
The results of Y-balance test scores. *Note:* Data are expressed as the mean ± standard deviation; “anterior,” “posteromedial,” “posterolateral,” and “composite” are normalized scores of the Y-balance test here. GSQ, good sleep quality; PSQ, poor sleep quality; ns, nonsignificant (*p* > 0.05); ⁣^∗^*p* < 0.05.

After controlling for potential confounding demographic variables (gender and age) and clinical variables (RMDQ, SAS, SDS, and FABQ), the PSQ group showed significantly poorer performance on OLS-C than the GSQ group (24.3 ± 18.9s vs. 50.8 ± 27.8s, *F* = 13.4, *p* < 0.001) (Figure 2). Likewise, the PSQ group demonstrated significantly lower scores in the posteromedial (106.8 ± 14% vs. 119.4 ± 14.3%, *F* = 4.9, *p*=0.029), posterolateral (108.2 ± 10.9% vs. 121 ± 12.5%, *F* = 10.0, *p*=0.002), and composite measures of the YBT (94.6 ± 9.9% vs. 104.8 ± 10.8%, *F* = 6.0, *p*=0.017) than the GSQ group after controlling for the aforementioned confounding variables (Figure 3). However, no significant between-group difference was noted in the anterior YBT score (68.7 ± 8% vs. 74 ± 10.4%, *F* = 0.5, *p*=0.466).

### 3.3. Regression Analysis

Sleep quality (PSQ/GSQ) emerged as the independent predictor of OLS-C scores, normalized posteromedial, posterolateral, and composite scores on the YBT, explaining 24.9%, 16.9%, 23.3%, and 20% of the variance, respectively (Table 2). In addition to sleep quality, age accounted for an additional 13.2% of the variance in OLS-C scores, 7.4% in normalized posteromedial scores, 5.2% in normalized posterolateral scores, and 7.5% in normalized composite scores of the YBT. Regarding the normalized anterior YBT scores, RMDQ and SAS scores independently accounted for 11.3% and 5.5% of its variance, respectively.

## 4. Discussion

This is the first study to investigate the influence of sleep quality on balance control in individuals with CLBP. Compared to those with CLBP alone, individuals with concomitant CLBP and PSQ exhibited inferior static and dynamic balance control, as measured by the OLS-C and YBT, respectively. Additionally, sleep quality accounted for 16.9%–24.9% of the variance in the static and dynamic balance performance, while age explained an additional 5.2%–13.2% of the variances. Furthermore, physical disability and anxiety emerged as potential factors associated with suboptimal dynamic balance control.

Our study revealed that PSQ was significantly associated with elevated levels of physical disability, anxiety, depression, and fear avoidance beliefs in individuals with CLBP, which aligns with prior literature [12, 13]. A systematic review and meta-analysis have shown that insufficient sleep affects both physical and mental health [51]. Notably, individuals with PSQ are more likely to experience impaired agility, mobility, and lower limb function, which can contribute to increased physical disability [52, 53]. Mental health conditions, such as anxiety and depression, have been identified as confounders in the bidirectional association between sleep quality and CLBP [9, 11, 54]. Sleep deprivation can induce or exacerbate anxiety and depression, while these conditions, characterized by hyperarousal, excessive worrying, and negative thought patterns, can also disrupt sleep initiation and maintenance [55]. Furthermore, PSQ has been linked to higher levels of rumination and repetitive negative thinking [56, 57], which are key components of fear avoidance beliefs [58, 59]. Therefore, it is essential to note that individuals with concomitant CLBP and PSQ commonly experience heightened levels of physical disability, anxiety, depression, and fear avoidance beliefs, which can, in turn, affect balance control.

After considering the aforementioned confounding factors, our results showed that PSQ might have a detrimental effect on both OLS-C and YBT performance in individuals with CLBP. This concurs with previous studies on sleep deprivation in healthy individuals, which found that suboptimal static balance (characterized by increased center of pressure displacement during quiet bipedal standing with eyes open on a force plate) could occur after sleep deprivation over two consecutive nights [15, 17, 19]. Our findings on YBT performance were similar to those of Tanwar et al. (2021), who reported that young adults with PSQ exhibited worse normalized composite scores compared to their GSQ counterparts [25]. However, our study found no significant difference in the normalized anterior scores of the YBT between the two groups. The discrepancy may be attributed to the low sensitivity of the YBT in detecting balance deficits in the anterior direction compared to the posteromedial or posterolateral directions, possibly due to visual compensation [60, 61]. Moreover, the association between PSQ and increased fall risk has been documented in patients with fibromyalgia [62], reinforcing the broader relevance of our findings across chronic pain conditions. These results suggest that improving sleep quality could be important for managing balance and preventing falls in individuals with CLBP and other chronic pain conditions.

PSQ is associated with impaired sensorimotor coupling, fatigue, psychological distress (e.g., anxiety and depression), and cognitive deficits (e.g., executive function and attention) in individuals with CLBP, potentially compromise balance control [9, 26, 63–66]. For example, inadequate sleep has been linked to attention impairments, which can reduce available attentional resources and delay reaction times, thereby affecting balance control [64, 67]. Aguiar and Barela (2015) reported that individuals with PSQ faced challenges in uncoupling irrelevant sensory information and were inefficient at selecting the most relevant sensory cues for appropriate motor responses in balance control, suggesting a noisy balance control system [66]. Furthermore, sleep disturbance may induce neurophysiological alterations, such as reduced muscle sympathetic nerve activity and delayed efferent signals from the central nervous system to balance control effectors in both healthy individuals and those with fibrositis syndrome [67–69]. Studies have also demonstrated that sleep deprivation decreases activity in brain regions related to the vestibular system (temporal lobes), proprioceptive system (basal ganglia and cerebellum), and attention and executive function (prefrontal cortex) in healthy individuals. These brain changes may increase the risk of falls and balance impairments [16–21]. Additionally, Thomas et al. revealed that one night of sleep deprivation resulted in decreased neuronal activity in the corticothalamic network, which is involved in attention and higher-order cognitive performance [70]. Given our results, future studies should investigate the central mechanisms underlying balance control impairments in individuals with concurrent CLBP and PSQ using neuroimaging techniques, such as electroencephalography or functional near-infrared spectroscopy [18].

Our findings indicate that sleep quality (16.9%–24.9%) is a significant determinant of impaired balance control in individuals with CLBP, followed by age (5.2%–13.2%). These results align with Liu-Ambrose et al., who found that age was the primary determinant of balance control, accounting for 9%–14% of the variance in older women with osteoporosis [50]. Emerging evidence supports the notion that improving sleep quality can reduce pain in various chronic pain conditions, including CLBP and chronic widespread pain [8, 71–74]. Afolalu et al. found that enhanced sleep quality was associated with improved physical function in the general population, although the effect size was small [51]. Therefore, to enhance balance control in adults with CLBP, it is crucial to identify and address those modifiable factors (sleep quality, physical disability, and anxiety) [75, 76]. However, further research, including controlled clinical trials, is necessary to validate these findings.

Several limitations should be considered when interpreting our results. First, our cross-sectional design precludes the establishment of causal relationships between sleep quality and balance control [77]. Second, our assessment of sleep quality relied on a self-reported questionnaire, although the psychometric properties of the PSQI have been established [9, 77, 78]. Future studies should incorporate objective measures such as polysomnography or actigraphy to collect objective data on sleep quality and quantity [77–79]. Third, this study did not include asymptomatic controls for comparison. Nevertheless, prior studies have established that individuals with CLBP generally exhibit poorer balance control than healthy control across adult populations [80–82]. Fourth, most of our participants were young, with an average age of 33.2 ± 12.5 years, and these findings may not be generalized to older adults with CLBP, who may be more susceptible to falls. Future studies should investigate balance control in older adults with concomitant CLBP and PSQ.

## 5. Conclusion

This study makes a pioneering contribution, by revealing the association between poorer sleep quality and impaired balance control in individuals with CLBP. PSQ, along with physical disability and anxiety, is a potential factor that contribute to impaired balance control in individuals with CLBP. Addressing sleep quality and managing these factors may be crucial for improving balance control and overall well-being in this population. Further research is warranted to investigate the effects of interventions targeting these modifiable factors on balance control outcomes.

## Figures and Tables

**Table 1:** Demographic and clinical characteristics.

	Poor sleep quality (*n* = 48)	Good sleep quality (*n* = 37)	*χ* ^2^/*t*/*z*	*p*
Gender				
Male	17	20	2.952	0.086
Female	31	17		
Age (years)	35.6 ± 13.5	29.9 ± 10.2	−2.213	0.03
Height (cm)	167.8 ± 9.2	167.6 ± 8.2	−0.092	0.927
Weight (kg)	64.6 ± 12.9	65.6 ± 13.6	0.348	0.728
BMI (kg/m^2^)	22.8 ± 2.9	23.2 ± 3.7	−0.31^†^	0.756
Left leg length (cm)	85.9 ± 5.7	84.5 ± 4.9	−1.204	0.232
Right leg length (cm)	85.7 ± 5.8	84.4 ± 5.1	−1.069	0.288
Pain duration (years)	7 ± 6.6	6.8 ± 6.3	−0.129^†^	0.898
Average pain	3.8 ± 1.3	3.5 ± 1.2	−1.079^†^	0.280
Current pain	3.2 ± 1.6	2.6 ± 1.4	−1.596^†^	0.111
Maximum pain	5.3 ± 1.6	4.9 ± 1.5	−1.201^†^	0.230
PSQI	9.2 ± 3	3.7 ± 1.4	−7.915^†^	< 0.001⁣^∗^
RMDQ	7.8 ± 5.7	4.4 ± 3.5	−2.972^†^	0.003⁣^∗^
SAS	46.9 ± 8.9	36 ± 5.2	−5.683^†^	< 0.001⁣^∗^
SDS	46.8 ± 10.7	34.5 ± 5.8	−5.384^†^	< 0.001⁣^∗^
FABQ (total score)	41.9 ± 10.8	32.5 ± 12.4	−3.385^†^	< 0.001⁣^∗^
FABQ-P	13.5 ± 4.3	10.6 ± 5.5	−2.445^†^	0.014⁣^∗^
FABQ-W	28.4 ± 8.6	21.9 ± 8.4	−3.087^†^	0.002⁣^∗^

*Note:* The values are presented as number or mean ± standard deviation.

Abbreviations: BMI, body mass index; FABQ, Fear Avoidance Beliefs Questionnaire; FABQ-P, Fear Avoidance Beliefs Questionnaire for physical activities; FABQ-W, Fear Avoidance Beliefs Questionnaire for work activities; PSQI, Pittsburgh Sleep Quality Index; RMDQ, Roland Morris disability questionnaire; SAS, Zung Self-Rating Anxiety Scale; SDS, Zung Self-Rating Depression Scale.

^†^Mann–Whiney *U*-tests.

⁣^∗^*p* < 0.05.

**Table 2:** Regression models^a^ summaries for OLS-C and normalized YBT scores including standardized beta coefficients and *R*^2^.

Variable	Model	Predictor	Standardized beta	*R* ^2^	*R* ^2^ change
OLS	1	Group	−0.499	0.249	
	2	Group	−0.413		
		Age	−0.373	0.381	0.132

Anterior	3	RMDQ	−0.337	0.113	
	4	RMDQ	−0.263		
		SAS	−0.244	0.168	0.055

Posteromedial	1	Group	−0.411	0.169	
	2	Group	−0.348		
		Age	−0.278	0.243	0.074

Posterolateral	1	Group	−0.482	0.233	
	2	Group	−0.428		
		Age	−0.236	0.285	0.052

Composite	1	Group	−0.448	0.2	
	2	Group	−0.384		
		Age	−0.281	0.275	0.075

Abbreviations: FABQ, Fear Avoidance Beliefs Questionnaire; OLS-C, one-leg stance with eyes closed; RMDQ, Roland Morris Disability Questionnaire; SAS, Self-Rating Anxiety Scale; SDS, Self-Rating Depression Scale; YBT, Y-balance test.

^a^Variables entered into the forward stepwise regression models: group (poor/good sleep quality), gender (female/male), age in years, RMDQ, SAS, SDS, and FABQ scores.

## Data Availability

Deidentified individual participant data can be made available upon request to wangxueqiang@sus.edu.cn.
